# Differences in patients derived from otolaryngology and other specialties with sleep apnea

**DOI:** 10.1186/s40463-019-0373-4

**Published:** 2019-10-22

**Authors:** Constanza Salas, Jorge Dreyse, Andrea Contreras, Gonzalo Nazar, Constanza Astorquiza, Rodrigo Cabezon, Gonzalo Labarca, Jorge Jorquera

**Affiliations:** 1 Centro de Enfermedades Respiratorias y grupo de estudio trastornos respiratorios del sueño (GETRS), Clínica Las Condes, Las Condes, Chile; 2 Neurología y grupo de estudio trastornos respiratorios del sueño (GETRS), Clínica Las Condes, Las Condes, Chile; 3 Otorrinolaringología y grupo de estudio trastornos respiratorios del sueño (GETRS), Clínica Las Condes, Las Condes, Chile; 4 Facultad de Medicina, Universidad San Sebastian, Lientur 1457, Concepcion, Chile

**Keywords:** Sleep apnea, obstructive, Sleep apnea syndrome, Otolaryngology

## Abstract

**Background:**

Snoring is a main concern in patients who consult an otolaryngologist (ENT physicians) and patients who have cardiovascular comorbidities or excessive daytime sleepiness who usually consult with other specialists. The aim of this study was to describe the clinical differences in patients with obstructive sleep apnea (OSA) referred from ENT or other specialists**.**

**Methods:**

A prospective study was carried out between June 2015 and July 2018 in a tertiary center. We included patients with suspected OSA referred by the Home Sleep Apnea Test (HSAT) from different specialties such as ENT or other specialties. The main outcome measures of our study were demographic characteristics, clinical characteristics, sleep questionnaire results and HSAT results between OSA patients referred from ENT or other specialists. We used a t-test and chi-squared test for analysis. The diagnostic accuracy of the sleep questionnaires was achieved using receiver operating characteristic (ROC) curve and the area under the curve (AUC).

**Results:**

A total of 481 patients were included. OSA was occurred in 82.4% of the subjects (90 in ENT and 306 in other specialties). Patients with OSA referred from other specialists were older than ENT patients (55 ± 13 vs 44 ± 12; *p* < 0.001), there was more obesity (IMC 31 ± 5.0 vs 28.7 ± 3.8; p < 0,001), a larger neck circumference (42.2 cm ± 3.7 vs 40.6 cm ± 3.0; p < 0.001) and more reported comorbidities (p < 0.001). ENT patients reported mild OSA (46% vs 31%, *p* = 0.015) and more positional apnea (62% vs 39%, *p* = 0.002). In this group, the STOP-BANG questionnaire showed an AUC 0.695 vs AUC 0.804, and for sensitivity, the best cutoff was 4 points.

Patients referred from otorhinolaryngology are different from those referred from other specialties. Clinical evaluation and screening of OSA should be patient-centered according to these clinical findings.

## Background

Obstructive sleep apnea (OSA) is a common condition with an estimated prevalence of 20% in the adult population worldwide [1–3]. In Chile, according to data from the national health survey in Chile, the population at risk for OSA is 31.2% and the risk of moderate/severe OSA is 8.9% [4]. OSA is linked with an increased risk of hypertension [5], traffic accidents [6, 7], metabolic disorders such as Type 2 diabetes mellitus, insulin resistance and dyslipidemia [8], and a greater number of both fatal and nonfatal cardiovascular events [9, 10]. Due to its complexity, it requires multidisciplinary management including both physicians and nonphysician practitioners from different specialties. Overall, therapeutic options are weight loss, postural therapy, myofunctional therapy, a mandibular advancement device, nasal surgery, multilevel surgery and continuous positive airway pressure devices (CPAP) that are chosen depending on different factors such as the severity of the disease, comorbidities, symptoms and anthropometric variables [3, 11, 12]. However, patients tend to consult specialist physicians who are often not associated with a sleep center and ignore their diagnosis based on their symptoms, personal experiences and recommendation of their acquaintances [13]. We believe people will consult an otolaryngologist (ENT physician) if snoring is their main concern or another specialist if their main problem is cardiovascular comorbidities or excessive daytime sleepiness. This may mean that patients who consult ENT specialists have different clinical characteristics than patients who are evaluated by other specialists (pulmonary physician, neurologists, and internal medicine specialists). The objective of this study was to describe the clinical and anthropometric characteristics, home sleep apnea test (HSAT) scores, and the accuracy of different diagnostic questionnaires in patients with sleep apnea according to the specialty of the doctor who referred them to the study (ENT or other specialties).

## Methods

### Participants

We performed an observational, prospective study between June 2015 and July 2018 in which all adult patients who were referred to a sleep study performed an ambulatory HSAT because of clinical suspicion of OSA (snoring symptoms, apneas observed by bed partner or excessive daytime sleepiness) in a sleep unit located in a tertiary center placed in Santiago, Chile. Our unit received referrals from both ENT and other medical specialists. Before to sleep study, patients were evaluated using a symptom standardized questionnaire with the purpose of evaluating their sleep schedule, degree of daytime sleepiness, snoring, apnea observed by a bed partner, insomnia, episodes of nocturnal suffocation, nycturia, morning headache, dry mouth upon awakening, memory loss and cognitive deterioration. Sociodemographic data, habits, comorbidities and anthropometric data (such as weight, mallampati score, height and neck, abdominal and waist circumferences) were recorded. The following data were collected: demographic, anthropometric, and comorbidity characteristics, different variables of the HSAT, the apnea-hypopnea index (AHI), mean and minimum oxygen saturation, the oxygen desaturation index (ODI), total sleep time with oxyhemoglobin saturation below 90% (TST-90%), and total time of recording. In addition, the Epworth sleepiness scale [14], modified Thornton scale [15], STOP-BANG questionnaire [16], Beck depression inventory [17], nasal obstruction symptom evaluation (NOSE) scale [18] and the Flemons predictive model score (adjusted neck circumference) [19] were calculated. The presence of OSA was defined as AHI ≥ 5 ev/hr. according to current recommendations [20], and the patients were classified according to the specialty of the referring doctor for subjects referred by ENT specialists and those referred by other specialists (pulmonary physicians, neurologists, and internal medicine specialists).

### Home sleep apnea test (HSAT)

To carry out the HSAT, Embletta MPR equipment ([Embla Systems Natus sleep products, USA) was used following the current recommendations and requirements of scientific societies for level III studies [20, 21]. The studies were conducted by the patients at their homes after previous instruction in the laboratory for installation by the patient or their families. They were given a printed user’s manual with iconographic information regarding the installation procedure. All recordings were downloaded the next day. The recordings had to meet the standards required by the American Academy of Sleep Medicine (AASM) for diagnostic studies; otherwise, it had to be repeated. The HSAT analysis was performed manually, according to the AASM guidelines [21], by a respiratory disease specialist with extensive experience in the diagnosis of sleep-disordered breathing (author J.J) who did not know the clinical history, the precedence, and the results of the sleep questionnaires analysis. The following definitions were used according to current recommendations. Apnea was defined as the absence of air flow for more than 10 s; obstructive apnea was defined as the absence of oronasal flow in the presence of abdominal thoracic movements; central apnea was defined as the absence of oronasal flow and abdominal thoracic movements; mixed apnea was defined as the absence of oronasal flow that begins with a central component and ends as obstruction; and hypopnea was defined as discernible airflow reduction (more than 30% and less than 90%) for a period greater than 10 s. To calculate the total number of respiratory events, we used the AHI, dividing the total events (apneas + hypopnea)/total time of recording in hours. A diagnosis of OSA was achieved when the AHI was greater than or equal to five events per hour.

### Statistical analysis

The results were expressed as the mean value ± standard deviation for the variables measured in numerical scales, as the median plus interquartile range 25–75 for the ordinal variables and the number and percentage for the ones measured in a nominal scale. The qualitative variables were compared using a Chi squared test, the ordinal variables were analyzed with a Mann-Whitney U test, and the continuous variables were assessed with Student’s t-test. The results of the HSAT were used as the gold standard. The ability of the different surveys (STOP-BANG, Thornton, and Epworth, Flemons predictive model and neck circumference) were used to discriminate patients with and without sleep apnea (AHI ≥ 5). Receiver operating characteristic (ROC) curves and degree or measure of separability was evaluated trough area under the curve (AUC) that was considered > 0.7 as good indicator of separability. The one with the highest amount of sensitivity and specificity for each of the questionnaires was established as the best cutoff point using the Youden Index. Data analysis and recording were performed using Excel 2016 software and SPSS 15.0 software (SPSS Inc., Chicago). A value of *p* ≤ 0.05 was considered as statistically significant.

## Results

### General characteristic and reason for referral between groups

A total of 481 adult subjects with clinical suspicion of OSA were evaluated consecutively with the HSAT. A total of 124 patients were derived by ENT compared to 357 by other specialists (pulmonology: 67.2%, cardiology: 13.72%, primary care: 4.0%, internal medicine: 4.88%, neurology: 3.31%, geriatrics: 1.29% and others: 5.6%). Reason for referral in the ENT group was: (Frequent snoring (≥ 1 times/week): 94.36%; usual snoring (≥ 5 times/week) 31.66%; bothered snoring (family group): 62.19%. and excessive daytime sleepiness (49%). Meanwhile in other specialties, reason for referral was: refractory hypertension or non- dipper hypertension (67.28%); snoring and observed apnea (65.90%); snorers with cardiovascular risk factors (8%) and regular snorer in subjects with metabolic disease (8%). The average age was 51.1 ± 14.7 years old, 81% of the patients were men, average neck circumference (NC) was 41.3 ± 3.9 cm, and the body mass index (BMI) was 29.8 ± 4.9 kg/m^2^. Of all subjects (396/481), 82.4% had OSA (AHI ≥ 5 ev/hr), 34.3% were mild, 34.1% were moderate, and 31.6% were severe. Table 1 describes the clinical characteristics and HSAT of patients with OSA derived by ENT and other specialties.

**Table 1:** Clinical features between obstructive sleep apnea

	ENT			SDB			
Variable	No OSA (n: 34)	OSA (n:90)	*p* value	No OSA (n:51)		OSA (n:306)	p value
Demography							
Age, years	40.7 ± 12.43	46.90 ± 11.98	0.002*	50.35 ± 16.39		55.42 ± 13.45	0.014*
Neck circumference, cms	38 ± 3.36	40.20 ± 3.46	<0.001*	38.77 ± 4.28		41.77 ± 3.64	<0.001*
Body mass index, kg/m^2^	26.13 ± 2.86	28.65 ± 3.99	<0.001*	27.89 ± 3.27		30.57 ± 4.49	<0.001*
Systolic pressure, mmHg	114 ± 13.77	119.59 ± 12.02	0.015*	123. 37 ± 16.29		123.61 ± 13.77	0.765
Diastolic pressure, mmHg	72.60 ± 9.15	77.03 ± 8.53	0.025*	75.43 ± 10.57		81.52 ± 9.72	0.002*
Pulse oximetry, %	96.33 ± 1.29	95.91 ± 1.36	0.232	94.91 ± 2.39		94.92 ± 1.30	0.471
Tobacco (Former- current)	48.9%	53.52%	0.423	62.74		58.73	0.524
Pack year index (Former - current)	10.37 ± 19.76	9.09 ± 11.03	0.321	22.35 ± 31.26		14.91 ± 18.08	0.042*
NOSE, (SD)	47.23 ± 25.62	42.11 ± 28.50	0.239	34.21 ± 25.24		34.15 ± 23.58	0.986
Rhinitis	51%	29.4%	0.005*	27.45%		25.5%	0.44
Hypertension	6.38%	21.17%	0.012*	23.52%		41.77%	0.008*
T2DM	0%	1.17%	0.613	1.96%		11.14%	0.023*
Insomnia	25.53%	18.82%	0.21	19.6%		28.53%	0.12
Sleep Questionnaires							
Epworth, (SD)	7.38 ± 4.51	7.84 ± 4.53	0.535	7.62 ± 4.36		8.16 ± 4.83	0.447
STOP-BANG, (SD)	3.08 ± 1.39	4.27 ± 1.43	<0.001*	3.45 ± 1.33		5.015 ± 1.42	<0.001*
Flemons, (SD)	41.70 ± 4.73	45.32 ± 6.79	0.001*	43.28 ± 5.22		47. 85 ± 6.89	<0.001*
Thornton, (SD)	2.13 ± 1.41	14.26 ± 5.65	<0.001*	2.56 ± 1.41		12.67 ± 5.06	<0.001*
OSA Severity							
Mild OSA		46%				31%	
Moderate OSA		30%				35%	
Severe OSA		24%				34%	

(OSA) and no OSA separated by Otolaryngology (ENT) and Sleep disorder breathing (SDB). NOSE: Nasal Obstruction and Septoplasty effectiveness Scale, AHI: Apnea hypopnea index, ODI: Oxygen desaturation index, SpO2: Oxygen saturation, TST-90%: Total sleep time with oxyhemoglobin saturation below 90%, T2DM: Type 2 diabetes mellitus. SD: Standard deviation. *Highlight: Statistically significant

### Differences in patients with OSA derived by ENT and other specialties

**Fig. 1:**
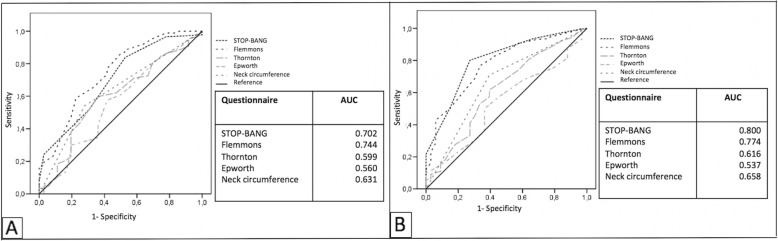
**a** ROC curve representing the accuracy of different questionnaires in otolaryngology patients, **b** ROC curve representing the accuracy of different questionnaires in other specialties patients. AUC: Area under the curve

A total of 90 patients with OSA were included in ENT group and 306 patients with OSA were included in other specialties group. When analyzing the differences between groups, the following were observed:

- Symptoms: There were no differences in the presence of symptoms, such as habitual snoring, apneas observed by bed partners, unrefreshing sleep or morning headache. No differences were observed regarding the depression symptoms or the use of hypnotics or antidepressants (Table 2).
- Anthropometric variables: patients referred by ENT specialists were younger (44.6 ± 12.4 vs 55.4 ± 13.4, *p <* 0.001), thinner (BMI 28.7 kg/m^2^ ± 3.8 vs 31.0 kg/m^2^ ± 5.0, *p* < 0.001) and had a smaller neck circumference (40.6 cm ± 3.0 vs 42.2 cm ± 3.7, *p <* 0.001) (Table 2).
- Comorbidities: Patients referred by ENT specialists had fewer comorbidities (hypertension, dyslipidemia, diabetes, coronary heart disease and gastroesophageal reflux), and there were fewer smokers in this group. No differences were observed regarding the prevalence of respiratory diseases, such asthma or chronic obstructive pulmonary disease or allergic rhinitis or nasal obstruction measured on the NOSE scale. Table 3 shows the associated comorbidities of the patients according to the referring physician.
- HSAT: Subjects with OSA referred by ENT specialists tended to present milder cases (46% v/s 31%, *p* = 0.015) and presented more episodes of strict positional apneas (62% v/s 39%, *p* = 0.002). In addition, they presented with fewer altered oximetric parameters (average saturation, minimum saturation and saturation time under 90%). Table 4 shows the main characteristics of HSAT for patients according to their referring doctor.
- Questionnaires: There was a difference in the predictive ability of the questionnaires to identify patients with OSA (Epworth, STOP-BANG, Thornton, Flemons predictive model and neck circumference) were different. Flemons predictive model achieved the best discrimination for OSA in patients referred by ENT specialists (AUC 0.744, *p* < 0.001) (Fig. 1a). STOP-BANG had the best discrimination for OSA in patients referred by other professionals (AUC 0.800, CI 0.766–0.882 versus AUC 0.702, CI 0.594–0.796, *p* = 0.001) (Fig. 1b). According to Youden index, the cutoff point that best discriminated OSA (AHI ≥ 5 ev/hr) in the ENT group was lower than in patients referred by other specialists, obtaining the best sensitivity and specificity for STOP-BANG with 4 and 5 points, for the Flemons predictive model with 45 and 47 points and for neck circumference with 41 and 42 cm, respectively (Table 5).

**Table 2:** Clinical and anthropometric comparison between ENT and Other specialties

	ENT (n: 90)	SDB (n: 306)	*P* value
Gender (male), (%)	88%	83%	0.327
Age, years (SD)	44.6 ± 12.4	55.4 ± 13.4	<0.001*
Body mass index, kg/m^2^ (SD)	28.7 ± 3.8	31.0 ± 5.0	<0.001*
Neck circumference, cms (SD)	40.6 ± 3.0	42.2 ± 3.7	<0.001*
Systolic Blood pressure, mmHg (SD)	119 ± 12	124 ± 13	0.001*
Diastolic Blood pressure, mmHg (SD)	78 ± 9	79 ± 11	0.337
NOSE, points	35 (10–60)	30 (15–50)	0.238
Pulse oximetry, % (SD)	95.8 ± 1.2	95.4 ± 1.9	0.076
Antidepressive drugs	23%	14%	0.072
Hypnotic use	8%	14%	0.198
Muscular relaxant use	3%	3%	0.718
Snorer	30%	28%	0.440
Apnea history	31%	37%	0.692
excessive daytime sleepiness	73%	68%	0.531
Headache	29%	39%	0.105

ENT: otolaryngology, NOSE: Nasal Obstruction and Septoplasty effectiveness Scale. SD: Standard deviation, *Highlight: Statistically significant

**Table 3:** Comorbidities assesment in both ENT and SDB groups

	ENT (n: 90)	Other specialties (n: 306)	*p*-value
Rhinitis	36%	33%	0.706
Tobacco (Former - current)	51%	57%	0.019*
Pack-year index, SD	11.5 ± 14.0	18.1 ± 28.1	0.121
Arterial Hypertension	24%	43%	0.001*
GERD	39%	51%	0.055
DM2	0%	10%	<0.001*
ACS	1%	10%	0.006*
Stroke	0%	4%	0.076
Asthma	4%	7%	0.471
COPD	0%	3%	0.207
Depression	9%	11%	0.697
Insomnia	13%	19%	0.272
Hypothyroidism	8%	15%	0.080
Dyslipidemia	27%	42%	0.010*
Mallampati			
I-II	20%	34.84%	0.07
III-IV	67.7%	60.85%	

*GERD* Gastroesophageal reflux disease, *DM* Diabetes Mellitus, *ACS* Acute coronary syndrome, *COPD* Chronic obstructive pulmonary disease, *ENT* Otolaryngology, *SD* Standard deviation,* Highlight: statistically significant

**Table 4:** Differences in Home sleep apnea test between ENT and SDB

	ENT (n: 90)	Other specialties (n: 306)	*p*- value
Home Sleep Apnea Test			
Total time, mins	436 ± 63	443 ± 79	0.443
AHI, ev/h	23.0 ± 19.7	27.4 ± 19.8	0.062
Average apnea time, seconds	18.0 ± 4.3	18.7 ± 3.7	0.111
ODI, ev/h	20.8 ± 18.6	24.5 ± 18.7	0.095
SpO_2_ average, %	92.9 ± 1.8	91.8 ± 2.9	0.001*
SpO_2_ minimum, %	81.6 ± 6.3	79.3 ± 9.2	0.029*
TST −90%, %	8.6 ± 14.2	16.2 ± 22.9	0.003*
Positional apnea	62%	39%	0.002*
Severity			0.035*
Mild OSA	46%	31%	
Moderate OSA	30%	35%	
Severe OSA	24%	34%	

*AHI* Apnea hypopnea index, *ODI* Oxygen desaturation index, *SpO*2 Oxygen saturation, *TST*-90% Total sleep time with oxyhemoglobin saturation below 90%, *OSA* Obstructive sleep apnea hypopnea syndrome, *ENT* Otolaryngology, *Highlights: Statistically significance

**Table 5:** Difference in questionnaire used in suspected OSA

	ENT (n: 90)	Other specialties (n: 306)	*p*- value
Epworth	8 (5–12)	7 (4–11)	0.890
STOP-BANG	5 (4–5)	6 (5–6)	<0.001*
Flemons	46 (43–48)	49 (46–52)	<0.001*
Thornton	13 (8–17)	13 (7–17)	0.736

Other specialties; *ENT* Otolaryngology, *Highlights: Statistically significance

## Discussion

The main results of this study were a) patients who consulted different specialists had different clinical characteristics; b) patients referred by ENT specialists were younger, thinner, had thinner necks, and they also had fewer comorbidities; however, they had the same symptoms of intensity of drowsiness and snoring; c) patients referred by ENT specialists had milder diseases and greater positional predominance; and d) regarding predictive models that achieved the best discrimination in patients with OSA, the predictive model of Flemons was the best for patients referred by ENT specialists, while STOP-BANG was the best for patients referred by other medical specialists.

To the best of our knowledge, this is the first study that evaluated the differences in the profiles of groups of patients studied for suspected sleep apnea referred by otolaryngology and other specialties. This is important because it can change how we appraise past and future studies on the OSA population. We demonstrate that patients who have OSA are different populations depending on which specialist they see for their issues and previous and future studies need to be re-evaluated for possible selection bias depending on which specialty published the data (physicians practicing sleep medicine or surgery). The decision that a patient initially consults an ENT doctor or other specialist depends on a number of sociocultural factors, personal or family experiences, and information in the media, among others. Patients could be expected to consult an ENT specialist if snoring is their main problem or other specialist if either cardiovascular comorbidities or excessive daytime sleepiness is their most relevant problems. However, in the present study, there were no differences in the reporting of habitual snoring, apneas observed by a bed partner, unrefreshing sleep, morning headache, excessive daytime sleepiness measured with the Epworth sleepiness scale or repercussions of snoring measured with the Thornton scale. Although the clinical history was similar, patients with OSA who consulted otolaryngologists were significantly younger and thinner. There were less smokers in that group, and there were fewer associated medical comorbidities commonly associated with OSA (hypertension, dyslipidemia, diabetes, coronary heart disease and gastroesophageal reflux) [8].

In addition, patients who consulted ENT specialists had a higher prevalence of mild disease (46% v/s 31%, *p* = 0.015), had more strict positional apnea episodes (62% v/s 39%, *p* = 0.002), and their oximetric parameters were less altered (average saturation, minimum saturation and TST- 90%). This is relevant when evaluating patients and the different perceptions that different specialists have regarding a patient with possible sleep apnea. Thus, despite the fact that the subjects presented with similar symptoms, they consulted different specialists, perhaps due to a perception of severity of the minor picture and the expectation of surgical resolution. According to previous publications, this is a novel finding; however, this finding is interesting because the initial approach of a patient who consults the ENT specialist compared to the approach of a patient who consults another specialist may be different and it might explain why different specialists present different perceptions of the same disease. This is relevant in order to define a potential “phenotype” of patients. Moreover, the performance on the different questionnaires is also different, with the Flemons predictive model achieving the best discrimination for OSA in patients referred by ENT specialists, while the STOP-BANG was the one that had the best discrimination for those referred by other professionals. In the ENT referred group, the best cut-off point to predict significant risk of sleep apnea using STOP-BANG as a screening method was 4 points versus 5 points in patients referred by other specialists. For the Flemons predictive model, the best were 45 and 47 points, and for neck circumference, the best were 41 and 42 cm, respectively. We showed that patients derived by ENT reported lowest neck circumference, age, neck circumference, and hypertension, (all variables included in both questionnaires). These variations can affect the ability of both STOP-BANG and Flemons questionnaires underestimating the predictive value in ENT group compared to other specialties.

These data are very relevant because the different models of clinical prediction have been evaluated in the general population in Chile [3, 22] and have not considered the potential selection bias created by the referral source or specialty performing the study. We did not use the Berlin questionnaire due to poor performance in our population [23]. This was a prospective study of consecutive patients with high n-value (481) and there was complete characterization of their diseases, major limitations, and possible bias that referring physicians held when carrying out an evaluation in a multidisciplinary center with established study programs. Finally, we used a HSAT or type III study as a reference standard; however, full polysomnography is better than HSAT for OSA diagnosis [24]. We decided to use HSAT due to recent publications showing that full polysomnography is not necessary for the management of patients with suspected OSA. Moreover, this approach is associated with potentially wasteful healthcare resources [3, 24].

## Conclusions

In conclusion, the patients with sleep apnea from different health teams were different. The subjects who consulted otolaryngologists were younger, thinner, with less comorbidities, but equally symptomatic. Therefore, the research strategies must be different and oriented to the requirements of the patients.

## Data Availability

All data will be available by personal communication with corresponding author.

## Abbreviations

AASM): American Academy of Sleep Medicine
AHI): Apnea-hypopnea index
AUC): area under the curve
BMI): Body mass index
CPAP): Continuous Positive Airway Pressure
ENT): Otolaryngology
HSAT): Home Sleep Apnea Test
NC): neck circumference
NOSE): nasal obstruction symptom evaluation
ODI): oxygen desaturation index
OSA): Obstructive sleep apnea
ROC): Receiver operating curves
TST-90%): Total sleep time with oxyhemoglobin saturation below 90%
